# Chemical Variability of the Leaf Essential Oil of Two Subspecies of *Juniperus oxycedrus* L. From Northwestern Algeria

**DOI:** 10.1002/cbdv.202401584

**Published:** 2024-11-09

**Authors:** Charaf Eddine Watheq Malti, Chahrazed Bekhechi, Khadidja Salhi, Brahim Babali, Joseph Casanova, Mathieu Paoli, Félix Tomi

**Affiliations:** ^1^ Faculté des Sciences et Technologies Université Ahmed Zabana Cité Bourmadia 48000 Relizane Algérie; ^2^ Laboratoire des Produits Naturels, Département de Biologie Université Abou Bekr Belkaïd, Imama 13000 Tlemcen Algérie; ^3^ Laboratoire d'Ecologie et Gestion des Ecosystèmes Naturels, Département d'Ecologie et Environnement Université Abou Bekr Belkaïd, Imama 13000 Tlemcen Algérie.; ^4^ Université de Corse-CNRS, UMR 6134 SPE Route des Sanguinaires 20000 Ajaccio France

**Keywords:** *Juniperus oxycedrus*, Northwestern Algeria, Leaf essential oil, Chemical composition, Statistical analysis

## Abstract

Thirty‐seven essential oil samples have been isolated from leaves of individual plants of *Juniperus oxycedrus* (subspecies *oxycedrus* and *macrocarpa*) growing wild in Tlemcen and Aïn Témouchent provinces (Northwestern Algeria). Analysis of eight selected oil samples by GC(RI), GC/MS and ^13^C NMR, allowed the identification of 88 components that accounted for 84.4–99.5 % of the whole compositions. The 37 compositions were subjected to statistical analysis which suggested the existence of groups and sub‐groups I, IIA and IIB. Group I, the most important group with 24 oil samples out of 37, differed from sub‐groups IIA and IIB, essentially by the high content of α‐pinene (mean value, 69.2 %; SD 6.24). Sub‐groups IIA and IIB were differentiated by their content of α‐pinene (Mean values, 49.7 % and 27.3 %, respectively) as well as δ‐3‐carene (10.3 %, sub‐group IIA), sabinene and (*Z*)‐6‐pentadecen‐2‐one (8.7 % and 4.9 %, respectively, sub‐group IIB).

## Graphical abstract

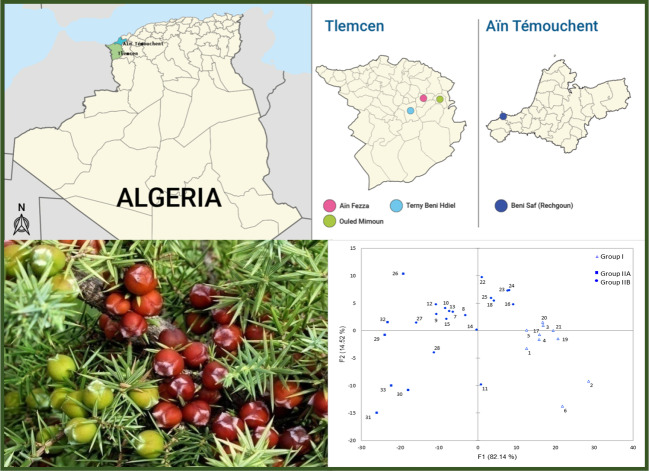

## Introduction

*Juniperus oxycedrus* L. (Cupressaceae family) chiefly a Mediterranean species, is an evergreen dioecious shrub. In the flora of Algeria, it is recognized three subspecies, including: subsp. *badia* (H. Gay) Deb., subsp. *macrocarpa* (S. & Sm.) Ball. and subsp. *oxycedrus* [Syn. subsp. *rufescens* (Link) Deb.].[^1^, ^2^] *J. oxycedrus* commonly known in Algeria as “Taga”,^[1]^ grows from North Africa (Morocco, Algeria and Tunisia), into Portugal, Spain, France, Italy, Greece, the Balkans, Turkey and eastward into Iran.^[3]^

Cade oil (empyreumatic oil) prepared by the destructive distillation of dry wood of *J. oxycedrus*, was largely employed as an antiparasitic and antiseptic for skin diseases, psoriasis and chronic eczema.^[4]^ This plant is widely used as traditional medicine. It is recommended against diabetes, diarrhea, dermatitis, rheumatism and eye infections.[^5^, ^6^, ^7^]

Oils of *J. oxycedrus* have many biological activities such as antibacterial,[^8^, ^9^, ^10^, ^11^] antifungal,^[8]^ antibiofilm,^[11]^ anti‐quorum sensing,^[12]^ antioxidant,[^12^, ^13^, ^14^] anti‐tyrosinase and anti‐cholinesterase.^[13]^

The composition of essential oil isolated from berries (fruits) of Algerian *J. oxycedrus* has been investigated. Most studies reported on the composition of a unique oil sample and concerned the subsp. *oxycedrus*.[^12^, ^15^, ^16^, ^17^] α‐Pinene (56.4–88.4 %) was the major component in three out of four reported compositions; followed by myrcene (6.7–10.4 %) and germacrene D (14.2 %) or α‐amorphene (9.0 %). The chemical composition of the fourth sample was dominated by germacrene D (38.3 %) and α‐pinene (17.7 %). More informative was the work recently published by our groups. Indeed, the chemical composition of 33 oil samples isolated from the berries of individual trees of two subspecies of *J. oxycedrus* L., collected in Northwestern Algeria, was investigated and submitted to statistical analysis. Groups and sub‐groups were distinguished with respect to their contents of α‐pinene, germacrene D, myrcene and manoyl oxide.^[18]^

In contrast, the composition of essential oil isolated from aerial parts (generally leaves and eventually stems) of Algerian *J. oxycedrus* subsp. *oxycedrus* and/or subsp. *macrocarpa* has been widely investigated although every report concerned only one oil sample.[^9^, ^11^, ^12^, ^13^, ^14^, ^19^, ^20^, ^21^, ^22^, ^23^, ^24^, ^25^, ^26^]

In six oil samples out of nine belonging to the *oxycedrus* subspecies, α‐pinene was the major component although its content varied drastically from 23.6 % to 56.1 %. Other components present at appreciable content were very different from sample to sample: β‐phellandrene,^[11]^ sabinene and terpinen‐4‐ol,^[14]^ δ‐3‐carene,^[19]^ germacrene D,^[20]^ bulnesol,^[13]^ epi‐manoyl oxide and (*E*,*E*)‐farnesol,^[14]^ abietadiene.[^12^, ^13^] The last three oil samples of the *oxycedrus* subspecies displayed very different compositions. The monoterpene‐rich oil sample from Mostaganem contained mainly terpinen‐4‐ol (17.48 %), α‐pinene (14.85 %) and sabinene (14.93 %).^[21]^ One oil sample from Tiaret Forest displayed germacrene D (27.41 %) δ‐3‐carene (24.73 %) and myrcene (13.85 %) as major components.^[22]^ In an oil sample from Tébessa various oxygenated pinane derivatives were detected, *trans*‐pinocarveol (7.0 %), *cis*‐verbenol (6.3 %), pinocarvone (5.1 %).^[23]^

Otherwise, α‐pinene (29.1 %), β‐pinene (17.6 %) and β‐copaene (19.3 %) were the major components of an essential oil isolated from leaves and fruits of *J. oxycedrus* ssp. *oxycedrus* harvested in Tiaret Forest.^[24]^ In contrast, another oil sample isolated from leaves, stems and fruits harvested in the Aures contained mainly manoyl oxide (23.4 %) beside (*Z*)‐pentadec‐6‐en‐2‐one (12.6 %).^[25]^

The composition of two oil samples of the subspecies *macrocarpa* has also been investigated. The composition of the former from El Kala) was dominated by germacrene D (21.3 %), followed by (*Z*,*Z*)‐farnesol (10.9 %).^[26]^ The major component of the sample from Oum El Bouaghi was the uncommon 5‐(Z)‐tetradecen‐1‐yl acetate (12.9 %).^[9]^

In a recent study, we evidenced a variability in the chemical composition of essential oils isolated from berries of *J. oxycedrus* (*oxycedrus* and *macrocarpa* subspecies) growing wild in provinces of Tlemcen and Aïn Témouchent, Northwestern Algeria.^[18]^ Statistical analyses suggested the existence of two groups which were distinguished on the basis of α‐pinene content. According to the amounts of α‐pinene, germacrene D and myrcene, as well as manoyl oxide, group II was divided into two sub‐groups. The chemical composition of the essential oils belonging to subsp. *oxycedrus* was dominated by monoterpenes. The main constituents were α‐pinene (23.7–63.2 %) with appreciable contents of germacrene D (4.5–25.4 %). In contrast, germacrene D (4.4–30.0 %) was the major component in oil samples of subsp. *macrocarpa*, accompanied by a lower content of α‐pinene (10.0–24.3 %) and a significant amount of manoyl oxide (4.3–9.9 %).^[18]^

The chemical variability of *J. oxycedrus* essential oil has been evidenced in a few countries and in a Mediterranean island:

– For instance, regarding essential oils isolated from 60 individual trees growing in Tunisia, the content of the major components varied substantially from sample to sample: α‐pinene (24.32–58.03 %), geranyl acetone (1.96–8.80 %), 13‐epi‐manoyl oxide (1.35–6.95 %). Hierarchical clustering and principal component analysis (PCA) allowed to establish four groups; one group being divided into two sub‐groups. The oils of the population from the continental site were clearly distinguished from those of the littoral localities^[27]^:
– The essential oil samples of *J. oxycedrus* collected among 20 populations across Bulgaria contained mainly α‐pinene, limonene, α‐curcumene, γ‐cadinene, δ‐cadinene, germacrene D, β‐caryophyllene, α‐caryophyllene, caryophyllene oxide and manoyl oxide, and they were grouped in eight chemotypes using advanced statistical methods^[28]^;
– The composition of 54 samples of leaf oil of *Juniperus oxycedrus* ssp. *oxycedrus* from Corsica was investigated. The main constituents were α‐pinene, β‐phellandrene and δ‐3‐carene. Principal component analysis, allowed the distinction of two compositions differentiated by the contents of α‐pinene, β‐phellandrene and δ‐3‐carene. In parallel, the chemical compositions of 18 leaf oil samples of *J. oxycedrus* ssp. *macrocarpa* have been investigated. The contents of the main components varied drastically from sample to sample: α‐pinene (28.7–76.4 %), δ‐3‐carene (up to 17.3 %), β‐phellandrene (up to 12.3 %), manoyl oxide (up to 8.1 %). Principal component analysis, as well as k‐means partition suggested a unique group, with few atypical samples. In the main group, α‐pinene (43.3–63.8 %) was accompanied by β‐phellandrene (up to 11.9 %), δ‐3‐carene (up to 7.5 %) and manoyl oxide (up to 4.5 %).^[30]^

Obviously, it appears from literature data that essential oil samples isolated from leaves (or aerial parts) of *Juniperus oxycedrus* displayed substantially different compositions. So, the aim of this study was to characterize the yield and the chemical composition of the essential oil from the leaves of *Juniperus oxycedrus* subsp. *oxycedrus* and subsp. *macrocarpa*, growing wild in Tlemcen and Aïn Témouchent provinces, Northwestern Algeria.

Firstly, we will report on the detailed analysis of eight essential oil samples, by combination of chromatographic and spectroscopic techniques. Secondly, the compositions of 37 leaf oil samples isolated from individual plants, were submitted to statistical analysis in order to evidence homogeneity or an eventual chemical variability.

## Results and Discussion

**Figure 1:**
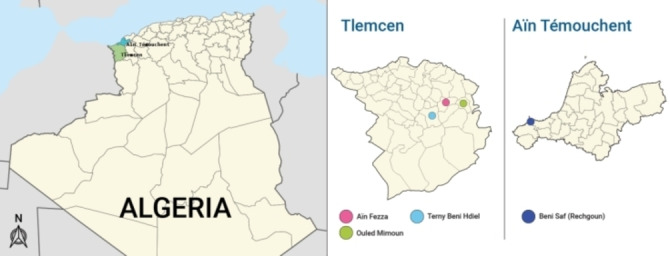
Sampling provinces of *Juniperus oxycedrus* from Northwestern of Algeria.

Leaves of 37 individual plants from *Juniperus oxycedrus* have been harvested in two provinces of Northwestern Algeria, Tlemcen (three locations, Ouled Mimoun, Terny and Aïn Fezza) and Aïn Témouchent (one location, Béni Saf) (Figure 1); two subspecies, subsp. *oxycedrus* (28 individuals) and subsp. *macrocarpa* (9 individuals). Leaves of individual plants have been hydrodistilled using a Clevenger‐type apparatus, yielding colorless essential oil samples. We will report first on the yield of essential oil depending of the subspecies and the location of harvest, then, on the detailed analysis of eight oil samples, selected on the basis of their chromatographic profiles, by GC in combination with retention indices on two chromatographic columns of different polarity, by GC/MS and by ^13^C NMR, without isolation of the individual components;[^31^, ^32^] lastly, on the statistical analysis of the 37 compositions.

### Essential Oil Yield

The yields of *J. oxycedrus* leaf essential oil (LEO) (w/w *vs*. dry material) varied substantially from sample to sample, even within subspecies, ranging between 0.01 to 0.20 % for the *oxycedrus* subspecies (28 samples) and from 0.02 % to 0.16 % for the *macrocarpa* subspecies (9 samples) (Table 1). The highest yields (0.11±0.07 %) were observed for the plants collected in Terny and the lowest (0.06±0.03 %) were observed for the plants harvested in Aïn Fezza, while similar yield mean values (0.09 % and 0.08 %) have been calculated for the plants harvested in Ouled Mimoun (*oxycedrus* subspecies) and Béni Saf (*macrocarpa* subspecies), respectively.

**Table 1:** Yields (%) of the essential oils isolated from leaves of two subspecies of *Juniperus oxycedrus*.

Locations of harvest	samples	Mean±SD^[a]^ (%)	Min^[b]^	Max^[c]^	Subspecies
Ouled Mimoun	9	0.09±0.05	0.04	0.19	*oxycedrus*
Terny	9	0.11±0.07	0.01	0.20	*oxycedrus*
Aïn Fezza	10	0.06±0.03	0.03	0.12	*oxycedrus*
Béni Saf	9	0.08±0.04	0.02	0.16	*macrocarpa*

[a] Standard deviation (SD); [b] Minimum (Min); [c] Maximum (Max)

### Chemical Composition of Selected Oil Samples

Eight oil samples, six of the subspecies *oxycedrus* and two of the subspecies *macrocarpa*, selected on the basis of their chromatographic profile, were submitted to gas chromatography (GC), in combination with retention indices (RIs) on two columns of different polarity, gas chromatography mass spectroscopy (GC/MS) and ^13^C nuclear magnetic resonance (NMR), following a computerized method developed at University of Corsica.[^31^, ^32^] The detailed analyses of those eight samples were reported in Table 2. In total, 88 compounds were identified, accounting for 84.4–99.5 % of the whole compositions, which were largely dominated by monoterpene hydrocarbons (68.6–83.9 %), sample S15 excepted (36.2 %). In seven oil samples, α‐pinene (43.7–72.1 %) was by far the major component followed by δ‐3‐carene (up to 12.8 %) and β‐phellandrene (up to 11.0 %). Myrcene (1.4–7.8 %), β‐pinene (0.9–4.1 %), sabinene (0.1–3.4 %) and *p*‐cymene (0.2–3.1 %) were found to be the other monoterpene hydrocarbons present at appreciable contents. Germacrene D (0.9–7.2 %) was the only sesquiterpene hydrocarbon present in a significant amount, beside γ‐cadinene (0.3–1.8 %), (*E*)‐β‐caryophyllene (0.2–1.1 %), δ‐cadinene (0.1–1.0 %) and α‐humulene (0.0–1.0 %). The occurrence of (*Z*)‐6‐pentadecen‐2‐one (0.1–1.8 %) could be highlighted. Conversely, the main components of sample S15 were α‐pinene (28.1 %) and germacrene D (15.7 %), followed by oxygenated sesquiterpenes, namely: 2,3‐dihydrofarnesol (3.6 % *vs*. 0.0–1.1 %), (2*E*,6*E*)‐farnesol (2.5 % *vs*. 0.3–2.2 %) and diterpenes, namely abietadiene (5.4 % *vs*. 0.2–1.8 %) and manoyl oxide (3.1 % *vs*. 0.0–0.9 %).

**Table 2:** Chemical composition of LEO of two subspecies of *J. oxycedrus*.

	Compounds^[a]^	RIa^[b]^	RIp^[b]^	RIaL^[c]^	RIpL^[c]^	S24 ^[d]^	S19 ^[d]^	S5^[d]^	S10^[d]^	S1^[d]^	S15^[d]^	S29 ^[e]^	S33^[e]^	Identification
1	Hexanal	772	1081	777	1082	0.2	0.2	0.6	0.1	1.3	0.4	0.2	‐	RI, MS, ^13^C NMR
2	(*E*)‐Hex‐2‐enal	823	1221	827	1216	0.2	0.1	0.3	0.1	1.3	0.3	0.5	0.2	RI, MS
3	Hexan‐1‐ol	846	1347	855	1351	Tr	‐	0.1	‐	0.4	‐	0.2	0.3	RI, MS
4	Tricyclene	919	nd	922	1012	0.2	0.2	0.3	0.2	0.1	0.1	0.2	0.1	RI, MS
5	α‐Thujene	921	1014	926	1027	0.1	Tr	0.1	Tr	Tr	0.1	0.8	0.5	RI, MS
6	α‐Pinene	930	1014	935	1025	72.1	62.3	61.7	57.4	48.8	28.1	67.0	43.7	RI, MS, ^13^C NMR
7	α‐Fenchene	940	1050	945	1061	0.1	Tr	0.7	Tr	Tr	0.1	0.1	0.5	RI, MS, ^13^C NMR
8	Camphene	942	1062	947	1069	0.4	0.3	0.3	0.3	0.4	0.1	0.3	0.2	RI, MS
9	Verbenene	945	1124	946	1124	0.2	0.3	0.5	0.2	0.2	0.1	0.1	0.3	RI, MS, ^13^C NMR
10	1‐Octen‐3‐ol	959	1444	966	1444	0.2	0.2	0.7	0.1	0.1	0.2	0.3	2.0	RI, MS, ^13^C NMR
11	Sabinene	963	1119	968	1122	0.3	0.2	0.2	0.2	0.1	0.3	2.7	3.4	RI, MS, ^13^C NMR
12	β‐Pinene	969	1109	973	1110	1.7	1.6	1.4	2.3	4.1	0.8	0.9	1.2	RI, MS, ^13^C NMR
13	Myrcene	979	1158	983	1161	1.5	1.4	1.4	2.3	3.7	0.7	7.8	2.1	RI, MS, ^13^C NMR
14	2‐Carene	994	1128	998	1134	0.1	0.4	0.2	0.6	0.5	0.1	0.1	0.1	RI, SM, ^13^C NMR
15	α‐Phellandrene	996	1162	999	1168	Tr	‐	0.3	0.4	0.7	0.1	‐	0.1	RI, MS, ^13^C NMR
16	δ‐3‐Carene	1004	1145	1007	1147	0.1	Tr	8.2	‐	Tr	3.0	0.6	12.8	RI, MS, ^13^C NMR
17	α‐Terpinene	1008	1177	1011	1178	Tr	‐	Tr	‐	‐	0.1	0.2	0.3	RI, MS
18	*p*‐Cymene	1011	1268	1015	1270	0.6	0.2	1.3	1.6	3.1	0.9	0.8	1.5	RI, MS, ^13^C NMR
19	Limonene*	1020	1198	1024	1198	1.0	1.3	1.0	1.7	1.9	0.5	1.4	1.1	RI, MS, ^13^C NMR
20	β‐Phellandrene*	1020	1208	1021	1209	1.6	0.3	3.5	6.3	11.0	0.6	0.1	1.2	RI, MS, ^13^C NMR
21	γ‐Terpinene	1047	1242	1050	1245	‐	‐	Tr	‐	‐	0.1	0.4	0.5	RI, MS
22	1‐Octanol	1051	1552	1057	1552	0.1	0.1	Tr	0.1	Tr	0.1	0.1	0.5	RI, MS, ^13^C NMR
23	*p*‐Cymenene	1071	1435	1074	1438	0.1	0.1	0.1	0.1	0.1	0.1	Tr	0.1	RI, MS
24	Terpinolene	1077	1280	1079	1282	‐	‐	0.3	0.3	0.2	0.3	0.4	1.4	RI, MS, ^13^C NMR
25	Linalool*	1082	1542	1086	1543	0.5	0.3	0.2	0.1	0.3	0.3	Tr	0.2	RI, MS
26	α‐Pinene oxide*	1082	1374	1085	1364	Tr	Tr	‐	Tr	Tr	Tr	0.1	‐	RI, MS
27	α‐Campholenal	1103	1487	1107	1496	0.2	0.4	0.6	0.2	0.2	0.2	0.2	0.3	RI, MS, ^13^C NMR
28	Fenchol*	1103	1583	1101	1570	0.2	0.2	0.1	0.2	0.1	0.1	0.2	0.3	RI, MS
29	Camphor	1118	1512	1125	1515	Tr	0.1	Tr	Tr	0.1	‐	‐	0.1	RI, MS
30	*trans*‐Pinocarveol	1121	1650	1126	1661	0.5	1.3	1.2	0.4	0.5	0.3	0.3	0.7	RI, MS, ^13^C NMR
31	*cis*‐Verbenol	1123	1650	1126	1660	‐	0.3	0.1	‐	‐	0.1	‐	0.3	RI, MS
32	*trans*‐Verbenol	1127	1673	1134	1680	0.3	1.2	0.7	0.3	0.2	0.2	0.2	1.3	RI, MS, ^13^C NMR
33	*p*‐Menth‐3‐en‐8‐ol	1133	1597	1140^f^	1600^f^	‐	‐	Tr	‐	‐	Tr	‐	‐	RI, MS
34	*trans*‐Pinocamphone*	1137	1511	1144	1523	0.1	0.6	0.5	Tr	0.7	0.1	0.3	0.4	RI, MS, ^13^C NMR
35	Pinocarvone*	1137	1564	1140	1576	0.1	0.3	0.4	0.2	0.2	0.1	Tr	Tr	RI, MS
36	*p*‐Mentha‐1,5‐dien‐8‐ol	1144	1721	1149	1725	0.1	0.1	0.3	0.1	0.1	0.1	0.1	0.4	RI, MS,
37	Borneol	1147	1695	1153	1700	‐	‐	0.1	Tr	0.1	‐	0.1	0.2	RI, MS
38	Cryptone	1154	1661	1157	1675	0.1	‐	0.4	0.4	0.5	Tr	‐	0.2	RI, MS
39	*p*‐Cymene‐8‐ol	1158	1844	1165	1848	‐	‐	0.4	0.2	0.1	0.1	0.1	0.4	RI, MS
40	Terpinen‐4‐ol	1160	1597	1165	1601	0.2	0.2	0.1	0.1	Tr	0.2	1.4	1.8	RI, MS, ^13^C NMR
41	Myrtenal	1168	1623	1171	1632	0.1	0.3	0.4	0.1	0.1	0.1	0.1	0.3	RI, MS,
42	α‐Terpineol	1170	1692	1176	1694	0.2	0.1	0.2	0.1	0.3	0.2	0.5	0.4	RI, MS, ^13^C NMR
43	Myrtenol	1177	1786	1182	1790	0.1	0.3	0.3	0.2	0.1	0.1	0.1	0.3	RI, MS
44	Verbenone	1183	1715	1184	1721	Tr	‐	0.1	‐	0.2	‐	Tr	0.1	RI, MS
45	*trans*‐Carveol	1195	1829	1201	1836	0.1	0.3	0.3	0.1	0.1	0.1	0.1	0.2	RI, MS
46	*cis*‐Carveol	1212	1865	1206	1854	0.1	0.1	0.1	0.3	0.2	Tr	0.1	0.1	RI, MS
47	Bornyl acetate	1267	1575	1270	1579	‐	‐	Tr	‐	0.3	0.3	0.1	0.3	RI, MS
48	α‐Terpinyl acetate	1329	1680	1333	1695	0.1	0.1	0.1	0.1	0.5	0.1	0.1	0.1	RI, MS, ^13^C NMR
49	α‐Cubebene	1345	1450	1352	1460	0.1	‐	Tr	Tr	Tr	0.1	0.1	0.1	RI, MS
50	α‐Ylangene	1367	1476	1370	1484	‐	‐	‐	‐	Tr	0.1	Tr	‐	RI, MS
51	α‐Copaene	1371	1484	1376	1491	0.1	‐	‐	0.1	Tr	0.1	0.1	Tr	RI, MS
52	β‐Elemene	1384	1583	1388	1591	Tr	0.1	Tr	0.2	0.1	0.2	0.1	0.2	RI, MS
53	(*E*)‐β‐Caryophyllene	1413	1589	1419	1599	0.6	0.9	0.2	1.1	0.6	1.7	0.4	0.2	RI, MS, ^13^C NMR
54	β‐Coapene	1422	1583	1427	1580	0.1	0.1	Tr	0.1	0.1	0.2	0.1	0.1	RI, MS
55	(*E)*‐β‐Farnesene	1443	1661	1449	1664	‐	‐	0.1	0.1	0.1	‐	0.1	‐	RI, MS
56	α‐Humulene	1446	1661	1449	1667	0.6	0.9	‐	1.0	0.4	1.4	0.4	0.1	RI, MS, ^13^C NMR
57	γ‐Muurolene	1467	1679	1473	1690	0.5	0.4	0.1	0.7	0.3	0.7	0.5	0.2	RI, MS, ^13^C NMR
58	Germacrene D	1472	1701	1476	1708	2.2	5.7	0.9	7.2	2.1	15.7	4.2	4.1	RI, MS, ^13^C NMR
59	2‐Tridecanone	1471	1801	1479	1808	0.6	0.4	0.2	0.2	0.2	‐	‐	0.1	RI, MS, ^13^C NMR
60	β‐Selinene	1477	1710	1481	1717	Tr	‐	‐	‐	Tr	Tr	Tr	Tr	RI, MS
61	α‐Muurolene	1489	1715	1491	1723	0.2	0.3	0.1	‐	0.2	0.5	0.2	0.2	RI, MS
62	β‐Bisabolene	1496	1719	1500	1728	Tr	‐	‐	Tr	0.1	0.2	‐	0.1	RI, MS
63	γ‐Cadinene	1501	1749	1506	1763	0.3	0.5	0.5	1.8	1.1	0.6	0.3	0.3	RI, MS, ^13^C NMR
64	Calamenene^[g]^	1505	1824	1510	1835	0.1	Tr	‐	0.1	Tr	0.1	Tr	Tr	RI, MS
65	δ‐Cadinene	1510	1749	1514	1756	0.7	0.6	0.1	0.7	0.3	1.5	0.8	1.0	RI, MS, ^13^C NMR
66	α‐Calacorene	1523	1907	1530	1921	‐	‐	‐	‐	Tr	Tr	Tr	0.1	RI, MS
67	α‐Cadinene	1526	1783	1527	1769	Tr	Tr	Tr	0.1	0.1	Tr	Tr	0.1	RI, MS
68	β‐Elemol	1530	2071	1537	2088	Tr	‐	‐	‐	‐	0.1	‐	0.4	RI, MS
69	Salviadienol	1534	2103	1545^[h]^	2110^[h]^	0.3	0.6	0.3	0.3	0.3	0.5	0.3	0.2	RI, MS
70	(*E*)‐Nerolidol	1546	2035	1550	2036	‐	‐	‐	‐	Tr	‐	‐	0.2	RI, MS
71	Caryophyllene oxide	1566	1972	1570	1986	0.3	0.6	0.3	0.2	0.3	0.4	0.1	Tr	RI, MS, ^13^C NMR
72	Salvial‐4(14)‐en‐1‐one	1573	1997	1585	2036	0.1	0.2	0.1	0.1	0.1	0.1	0.1	0.1	RI, MS
73	*cis*‐Guai‐6‐en‐10‐ol	1590	1988	1575^[i]^	2000^[i]^	0.4	0.9	0.3	0.4	0.4	0.7	0.3	0.2	RI, MS, ^13^C NMR
74	1,10‐di‐epi‐Cubenol	1610	2051	1606	2074	0.1	0.1	0.1	0.7	0.8	0.1	Tr	0.1	RI, MS
75	γ‐Eudesmol	1617	2163	1617	2176	Tr	0.2	0.1	Tr	0.1	0.1	0.1	Tr	RI, MS
76	τ‐Muurolol*	1620	2177	1631	2186	0.1	0.2	Tr	0.2	Tr	0.3	0.1	0.2	RI, MS
77	τ‐Cadinol*	1620	2160	1626	2170	Tr	0.1	0.1	Tr	0.2	0.1	0.1	0.1	RI, MS
78	β‐Eudesmol	1631	2219	1634	2223	‐	0.4	0.1	0.1	0.1	0.6	0.2	0.1	RI, MS
79	α‐Cadinol	1633	2221	1640	2227	0.2	0.4	Tr	Tr	0.1	0.4	0.3	0.7	RI, MS, ^13^C NMR
80	(*Z*)‐6‐Pentadecen‐2‐one	1647	2027	1647^[j]^	2030^[j]^	1.7	1.8	0.9	1.6	1.3	0.1	0.1	0.5	RI, MS, ^13^C NMR
81	Eudesma‐4(15),7‐dien‐1‐ol	1664	2345	1676	2371	0.1	0.1	0.1	0.1	0.2	0.3	0.2	0.1	RI, MS
82	2,3‐Dihydrofarnesol	1668	2260	1668^[k]^	2262^[l]^	0.5	1.1	0.3	0.3	0.6	3.6	‐	0.3	RI, MS, ^13^C NMR
83	(2*Z*,6*E*)‐Farnesal	1685	2310	1688^[m]^	2219^[m]^	0.1	0.2	0.1	0.2	0.1	0.6	‐	0.1	RI, MS, ^13^C NMR
84	(2*E*,6*E*)‐Farnesol	1697	2355	1710	2366	0.4	2.2	0.3	0.5	0.9	2.5	0.5	0.6	RI, MS, ^13^C NMR
85	(2*E*,6*E*)‐Farnesal	1711	2257	1715^[n]^	2264^[n]^	0.2	0.4	0.1	0.3	0.1	0.9	‐	0.2	RI, MS, ^13^C NMR
86	Manoyl oxide	1976	2329	1990	2376	‐	0.3	0.9	0.9	0.1	3.1	0.2	0.3	RI, MS, ^13^C NMR
87	Abietatriene	2028	2478	2033	2506	0.4	0.6	0.3	0.3	0.9	1.5	0.1	0.5	RI, MS, ^13^C NMR
88	Abietadiene	2064	2441	2062	2450	0.4	0.7	0.3	0.5	0.9	5.4	0.2	1.8	RI, MS, ^13^C NMR
	Monoterpene hydrocarbons					80.1	68.6	81.5	73.9	74.9	36.2	83.9	71.1	
	Oxygenated monoterpenes					3.0	6.2	6.6	3.1	4.9	2.7	4.1	8.4	
	Sesquiterpene hydrocarbons					6.1	9.9	2.2	13.4	5.7	23.1	7.3	6.9	
	Oxygenated sesquiterpenes					2.8	7.7	2.3	3.4	4.3	11.3	2.3	3.6	
	Diterpene hydrocarbons					0.8	1.3	0.6	0.8	1.8	6.9	0.3	2.3	
	Oxygenated diterpenes					0.0	0.3	0.9	0.9	0.1	3.1	0.2	0.3	
	Others					2.4	2.4	2.6	2.0	4.4	1.1	1.4	3.5	
	Total (%)					95.2	96.4	96.7	97.5	96.1	84.4	99.5	96.1	

[a] Components have been listed following their order of elution on apolar column (BP‐1); Percentages on apolar column, except those with an asterisk, % on polar column (BP‐20); [b] RIa, RIp: Retention indices on apolar and polar columns, respectively; [c] RIaL, RIpL: Retention indices from literature, on apolar and polar columns, respectively; Babushok *et al*., [33] otherwise stated; [d] Subsp. *oxycedrus*; [e] Subsp. *macrocarpa*; ^13^C NMR: Compound identified by ^13^C NMR, at least in one oil sample; nd: Not determined; Tr: Traces; [f] ref [34]; [g] Isomer not determined; [ h] ref [35]; [i] ref [36]; [j] ref [29], [k] ref [37]; [l] ref [38]; [m] ref [39]; [n] ref [40]

### Chemical Variability

Thirty‐seven essential oil samples were obtained by hydrodistillation of leaves collected from individual trees of *J. oxycedrus* in four locations of Northwestern of Algeria: Ouled Mimoun, Terny and Aïn Fezza (Tlemcen province, *oxycedrus* subspecies, 28 samples) and Béni Saf (Aïn Témouchent province, *macrocarpa* subspecies, 9 samples) (Figure 1).

**Figure 2:**
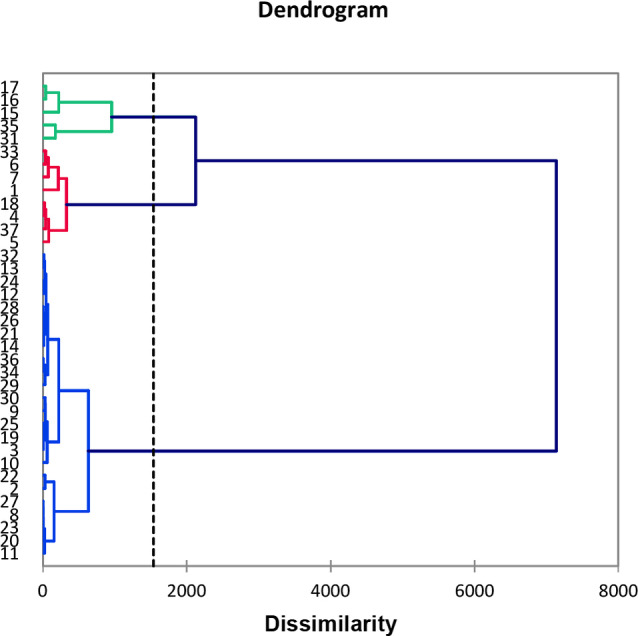
Hierarchical Cluster Analysis of the compositions of the 37 LEO of two subspecies of *Juniperus oxycedrus*.

**Figure 3:**
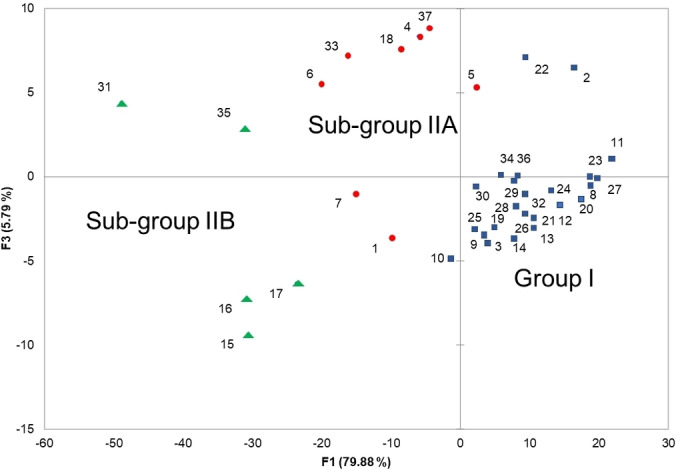
Discriminant analysis scatterplot of the oil constituents of 37 LEO of two subspecies of *Juniperus oxycedrus*.

The 37 compositions were subjected to statistical analysis in order to distinguish clusters. The combination of hierarchical clustering dendrogram (HCA, Figure 2), and principal component analysis (PCA; Figure 3), for which the two axes (F1 and F3) accounted for 85.7 % of the total variance of the population, suggested the existence of two principal groups, one of these being subdivided into two sub groups IIA and IIB.

Group I, by far the most important group with 24 oil samples out of 37, differed from sub‐groups IIA and IIB, essentially by the content of α‐pinene (69.2 %, mean value, SD 6.24; *vs*. 49.7 %, SD 7.27 and 27.3 %, SD 7.89) (Table 3). Otherwise, regarding major components of group I, α‐pinene was followed by germacrene D (M=4.2 %, SD 2.39).

**Table 3:** Chemical variability of *Juniperus oxycedrus* LEO.

Groups/samples	Group I/24				Group II/13							
Sub‐groups/samples					IIA/8				IIB/5			
Components^[a]^	M^[b]^	SD^[c]^	Min^[d]^	Max^[e]^	M^[b]^	SD^[c]^	Min^[d]^	Max^[e]^	M^[b]^	SD^[c]^	Min^[d]^	Max^[e]^
α‐Thujene	0.2	0.34	Tr	0.9	0.2	0.26	Tr	0.7	1.5	2.04	0.0	4.1
α‐Pinene	69.2	6.24	57.4	80.9	49.7	7.27	39.4	61.7	27.3	7.89	14.3	35.4
Oct‐1‐en‐3‐ol	0.2	0.14	0.0	0.5	0.8	0.59	0.1	2.0	0.3	0.17	0.2	0.6
Sabinene	0.9	1.40	0.2	5.3	1.0	1.52	0.1	3.5	8.7	11.94	0.1	25.2
β‐Pinene	1.6	0.42	0.9	2.4	1.7	0.98	1.2	4.1	0.9	0.10	0.8	1.1
Myrcene	1.9	1.33	0.7	7.8	1.9	0.83	1.1	3.7	1.3	0.74	0.7	2.4
δ‐3‐Carene	0.9	2.32	0.0	9.3	10.3	4.69	Tr	14.6	1.4	1.06	0.3	3.0
α‐Terpinene	0.1	0.18	0.0	0.7	0.1	0.11	0.0	0.3	0.7	0.99	Tr	2.2
*p*‐Cymene	0.5	0.41	0.1	1.6	1.5	1.00	0.1	3.1	2.8	2.82	0.6	4.6
Limonene	1.0	0.35	Tr	1.7	1.2	0.44	0.9	1.9	1.8	1.41	0.5	3.4
β‐Phellandrene	1.2	1.73	0.1	6.3	3.3	3.72	0.1	11.0	1.1	0.02	0.4	2.8
γ‐Terpinene	0.2	0.35	0.0	1.3	0.1	0.18	0.0	0.5	1.6	2.10	0.0	4.3
Terpinen‐4‐ol	0.5	0.78	Tr	2.8	0.6	1.00	Tr	2.6	4.2	5.42	0.2	11.6
Germacrene D	4.2	2.39	Tr	10.2	3.3	3.38	0.4	11.1	4.9	6.15	0.9	15.7
2‐Tridecanone	0.2	0.18	0.0	0.6	0.3	0.20	0.0	0.6	1.4	1.58	0.0	3.7
γ‐Cadinene	0.6	0.46	0.1	1.8	0.8	0.62	0.2	1.9	0.9	0.97	0.1	2.4
(*E*)‐Nerolidol	Tr	0.01	0.0	Tr	0.5	1.30	0.0	3.7	0.0	0.01	0.0	Tr
(*Z*)‐6‐Pentadecen‐2‐one	1.1	0.76	0.1	2.8	1.7	0.93	0.5	2.9	4.9	6.09	0.1	12.0
2,3‐Dihydrofarnesol	0.3	0.24	0.0	1.1	0.5	0.36	0.2	1.2	1.5	1.48	0.1	3.6
(2*E*,6*E*)‐Farnesol	0.8	0.66	Tr	2.5	0.7	0.46	0.2	1.7	1.9	0.73	1.1	2.7
Manoyl oxide	0.3	0.32	0.0	1.0	0.5	0.25	0.1	0.9	2.2	0.80	0.9	3.1
Abietadiene	0.5	0.43	0.0	1.9	1.0	0.81	0.1	2.5	2.4	2.06	0.7	5.4

[a] Percentages are given on apolar column (BP‐1); [b] Mean (M); [c] Standard deviation (SD); [d] Minimum content (Min); [e] Maximum content (Max); Tr: Traces.

Based on the contents of α‐pinene, sabinene, δ‐3‐carene, β‐phellandrene, terpinen‐4‐ol as well as 2‐tridecanone, (*Z*)‐6‐pentadecen‐2‐one, abietadiene and manoyl oxide, the samples of group II could be divided into two sub‐groups, IIA and IIB (Figure 3, Table 3).

Sub‐group IIA contained higher amount of α‐pinene (M=49.7 %, SD7.27) than sub‐group IIB (M=27.3 %, SD 7.89). Both sub‐groups differed mainly by their contents of sabinene (1.0 *vs*. 8.7 %), δ‐3‐carene (10.3 *vs*. 1.4 %) and (*Z*)‐6‐pentadecen‐2‐one (1.7 *vs*. 4.9 %).

It could be highlighted that all the samples from Aïn Fezza (S19‐S28, *oxycedrus* subspecies) belonged to group I. The nine samples from Ouled Mimoun (S1‐S9, *oxycedrus* subspecies) were equally reparteed between group I and sub‐group IIA. The nine samples from Terny (S10‐S18, *oxycedrus* subspecies) were reparteed between group I and sub‐group IIB, a last sample belonged to sub‐group IIA. Lastly, the nine samples from Béni Saf, *macrocarpa* subspecies, are mainly found in group I (5 samples) beside sub‐groups IIA (2 samples) and IIB (2 samples). The chemical variability appeared not related to the subspecies.

Comparing with previous results relative to the composition of *J. oxycedrus* leaf oil from Algeria, reported in the literature:

– an α‐pinene‐rich essential oil (92.2 %) was reported from National Park of Djurdjura, Bouira (Northern Central Algeria). This composition is clearly related to group I (highest content in α‐pinene)^[17]^;
– an essential oil sample from Batna, (Northeastern Algeria) containing α‐pinene, 56.1 % may be associated to sub‐group IIA, although it differed by a high content of β‐phellandrene (17.9 %)^[11]^;
– a composition from Tébessa, East Algeria with α‐pinene 42.2 % may be also associated to group IIA, although it differed by a high content of abietadiene (7.3 %)^[12]^;
– an oil sample from Laghouat (Central North Algeria) displayed a content of α‐pinene (37.6 %) similar to those of some oil samples of sub‐group IIA, however with higher content of abietadiene (8.3 %) and bulnesol (7.2 %, scarcely reported in *J. oxycedrus* essential oils)^[13]^;
– In parallel, a sample from Bouira (Central Algeria) contained α‐pinene (36.7 %) and it belongs to sub‐group IIA, taking into account the appreciable content of δ‐3‐carene (10.6 %)^[19]^;
– Most in the South, at Founassa, near Aïn Sefra, an essential oil sample contained fair amount of α‐pinene (33.4 %). However, the high content of germacrene D (23.7 %) differentiated this sample from ours of sub‐group IIB^[20]^;
– A first sample from Mostaganem (Northwestern Algeria), that contained α‐pinene as major component (23.6 %), followed with sabinene (10.8 %) may be associated to sub‐group IIB, although it contained fair amounts of farnesol (8.5 %) and epi‐manoyl oxide (11.5 %), reported for the first time in *J. oxycedrus* essential oils^[14]^;
– Another sample from Mostaganem, containing similar amounts of α‐pinene (14.9 %) and sabinene (14.9 %) could be also associated to sub‐group IIB, however, it differed by the content of terpinen‐4‐ol (17.5 %,)^[21]^;

Lastly, the compositions of two other Algerian *J. oxycedrus* oil samples look atypical with respect to those observed in Tlemcen and Aïn Témouchent provinces, reported here and they cannot be introduced in any of the groups and sub‐groups. For instance, the composition of an oil sample from Tiaret Forest (Northwestern Algeria), was dominated by germacrene D (27.4 %), δ‐3‐carene (24.7 %), accompanied by myrcene (13.9 %) beside α‐pinene (4.1 %).^[22]^ A sample from Tébessa (Eastern Algeria) contained mainly oxygenated pinane derivatives, *trans*‐pinocarveol (7.0 %), *cis*‐verbenol (6.3 %), pinocarvone (5.1 %), verbenone (3.6 %) while α‐pinene accounted for 0.2 % only.^[23]^

Concerning leaf oils from the subspecies *macrocarpa*, they could not associate with our groups and sub‐groups. Indeed, the first one from El Kala, contained mainly germacrene D (21.3 %), (*Z*,*Z*)‐farnesol (10.9 %) and 8,13‐epoxy‐14,15‐dinorlabdane (8.8 %).^[26]^ The second sample from Oum El Bouaghi, exhibited a very unusual composition dominated by (*Z*)‐5‐tetradecen‐1‐yl acetate (12.9 %) and γ‐muurolene (9.1 %).^[9]^

Comparing the present results with previous studies that included statistical analysis, the compositions of essential oil samples from group I (α‐pinene M=69.2 %, SD 6.24) and sub‐group IIA (α‐pinene M=49.7 %, SD 7.27) resembled to those of essential oils from Corsica (subspecies *oxycedrus*) with α‐pinene M=73.3 %, SD 5.8 (group I) and M=56.1 %, SD 5.7 (group II).^[29]^ Otherwise, we noticed that the nine samples from Béni Saf, *macrocarpa* subspecies, are present in the three groups and sub‐groups. Similarly, the chemical compositions of 18 leaf oil samples of *J. oxycedrus* ssp. *macrocarpa* from Corsica, varied substantially from sample to sample.^[30]^

Regarding the compositions of essential oils isolated from individual trees growing in Tunisia, α‐pinene was always the major component although its content varied substantially (24.32–58.03 %) as observed in the present study (14.3–80.9 %). α‐Pinene was accompanied by various compounds not present in our samples, β‐bourbonene (up to 14.28 %), 10‐epi‐α‐muurolol (up to 11.13 %), geranyl acetone (up to 8.80 %), 13‐epi‐manoyl oxide (up to 6.95 %). Four groups were distinguished, however, the mean value of every component in each group was not given.^[27]^

The essential oil samples of *J. oxycedrus* collected among 20 populations across Bulgaria contained various components with low to medium content: α‐pinene (6.5–24.8 %, much lower content than in our samples), manoyl oxide (4.9–18.4 %), limonene (1.8–16.7 %), caryophyllene oxide (0.7–13.2 %), β‐caryophyllene (0.1–12.5 %), δ‐cadinene (0.4–11.9 %), germacrene D (0.0–11.7 %). Samples were grouped in eight chemotypes (however, the mean percentage of every compound with respect to the chemotype, was not given).^[28]^

## Conclusions

Thirty‐seven essential oil samples have been isolated from leaves of *J. oxycedrus* (subspecies *oxycedrus* and *macrocarpa*) growing wild in Tlemcen and Aïn Témouchent provinces (Northwestern Algeria). Analysis by combination of chromatographic and spectroscopic techniques of eight selected oil samples allowed the identification of 88 components that accounted for 84.4–99.5 % of the whole compositions. α‐Pinene was the major component although its percentage varied substantially. The 37 compositions were subjected to statistical analysis which suggested the existence of two principal groups I and II, one of these being subdivided into two sub‐groups IIA and IIB. Group I, the most important group with 24 oil samples out of 37, differed from sub‐groups IIA and IIB, essentially by the high content of α‐pinene (mean value, 69.2 %; SD 6.24). Sub‐groups IIA and IIB were differentiated by their content of α‐pinene (Mean values, 49.7 % and 27.3 %, respectively) as well as δ‐3‐carene (10.3 %, sub‐group IIA) and sabinene (8.7 %, sub group IIB). The fair content of (*Z*)‐6‐pentadecen‐2‐one in sub‐group IIB (Mean value 4.9 %) could be noticed. The compositions of *J. oxycedrus* essential oils from Algeria, previously reported, were discussed with respect to groups and sub‐groups of the present study.

## Experimental Part

### Plant Material

Leaves from 37 individual plants of two subspecies of *Juniperus oxycedrus* were collected during December 2018 to March 2019 in two localities of Northwestern of Algeria (Figure 1). Twenty‐eight samples of *J. oxycedrus* subsp. *oxycedrus* were sampled at Tlemcen province (Ouled Mimoun: S1‐S9; Terny: S10‐S18; Aïn Fezza: S19‐S28) and nine samples of *J. oxycedrus* subsp. *macrocarpa* were collected at Aïn Témouchent province (Béni Saf: S29‐S37).

Identification of the plants was performed by Dr. B. Babali and Prof. F. Hassani (Laboratory of Ecology and Management of Natural Ecosystems, University of Tlemcen, Algeria). A voucher specimen has been deposited at the Laboratory of Natural Products (Department of Biology, University of Tlemcen) under the accession N°C. 48.

### Essential Oil Distillation

The leaves were dried for a week, in the shade, and then submitted (452–546 g) to hydrodistillation for 2 h using a Clevenger‐type apparatus. Yields have been calculated from dry material (w/w).

### Analytical GC

GC analyses were performed on a Perkin‐Elmer Clarus 500 gas chromatograph (FID) equipped two fused silica capillary columns (50 m×0.22 mm, 0.25 μm film thickness), BP‐1 (polydimethyl siloxane) and BP‐20 (polyethylene glycol). The oven temperature was programmed from 60 °C to 220 °C at 2 °C/min and then held isothermal at 220 °C for 20 min, injector temperature: 250 °C; detector temperature: 250 °C; carrier gas: hydrogen (1.0 mL/min); split: 1/60.

The relative proportions of the oil constituents were expressed as percentages obtained by peak area normalization, without using correcting factors. Retention indices (RI) were determined relative to the retention times of a series of *n*‐alkanes with linear interpolation (“Target Compounds” software from Perkin‐Elmer).

### GC/MS Analysis

The essential oils were analyzed with a Perkin‐Elmer TurboMass detector (quadrupole), directly coupled to a Perkin‐Elmer Autosystem XL, equipped with a fused‐silica capillary column (50 m×0.22 mm i.d., film thickness 0.25 μm), BP‐1 (dimethylpolysiloxane). Carrier gas, helium at 0.8 mL/min; split, 1/60; injection volume, 0.5 μL; injector temperature, 250 °C; oven temperature programmed from 60 °C to 220 °C at 2 °C/min and then held isothermal (20 min); Ion source temperature, 250 °C; energy ionization, 70 eV; electron ionization mass spectra were acquired over the mass range 40–400 Da.

### ^13^C NMR Analyses

^13^C NMR analysis was performed on a Bruker AVANCE 400 Fourier Transform spectrometer operating at 100.623 MHz for ^13^C, equipped with a 5 mm probe, in deuterated chloroform (CDCl_3_), with all shifts referred to internal tetramethylsilane (TMS). ^13^C NMR spectra were recorded with the following parameters: pulse width (PW), 4 μs (flip angle 45°); acquisition time, 2.73 s for 128 K data table with a spectral width (SW) of 220 000 Hz (220 ppm); CPD mode decoupling; digital resolution 0.183 Hz/pt. The number of accumulated scans ranged 2 000–3 000 for each sample (around 40 mg of oil in 0.5 mL of CDCl_3_). Exponential line broadening multiplication (1.0 Hz) of the free induction decay was applied before Fourier Transform.

### Identification of Components

Identification of the components was based on:

– comparison of their GC retention indices (RI) on polar and apolar columns, determined relative to the retention times of a series of *n*‐alkanes with linear interpolation (Target Compounds software of Perkin‐Elmer), with those of authentic compounds and with reference data^[33]^;
– computer matching against commercial mass spectral libraries[^41^, ^42^, ^43^];
– comparison of the signals in the ^13^C NMR spectra of essential oils with those of reference spectra compiled in the laboratory spectral library, with the help of a laboratory‐made software.[^31^, ^32^, ^44^] In the investigated samples individual components were identified by NMR at contents as low as 0.4–0.5 %.

### Data Analysis

Principal Components Analysis (PCA) and Hierarchical clustering (Ward's method) were performed by Xlstat (Adinsoft, France).^[45]^

## Author Contributions

Conceptualization: C.B., J.C. and F.T.; methodology: C.B. and F.T., sampling and extraction: C.E.W.M. and K.S. plant identification: B.B.; investigation: formal analysis, C.B. and M.P.; original draft preparation: C.B., J.C. and F.T., writing review: C.B. and J.C.

## Conflict of Interests

The authors declare no conflict of interest.

1

## Supporting information

As a service to our authors and readers, this journal provides supporting information supplied by the authors. Such materials are peer reviewed and may be re‐organized for online delivery, but are not copy‐edited or typeset. Technical support issues arising from supporting information (other than missing files) should be addressed to the authors.

Supporting Information

## Data Availability

The data that support the findings of this study are available from the corresponding author upon reasonable request.
